# *Neospora caninum* inhibits tumor development by activating the immune response and destroying tumor cells in a B16F10 melanoma model

**DOI:** 10.1186/s13071-022-05456-8

**Published:** 2022-09-23

**Authors:** Xiaojin Li, Meng Qi, Kai He, Haiyan Liu, Wenlan Yan, Lizhuo Zhao, Yanyan Jia, Lei He, Chaochao Lv, Min Zhang, Zhiguo Wei, Wenchao Yan, Tianqi Wang, Fuchang Yu, Weifeng Qian

**Affiliations:** 1 College of Animal Science and Technology, Henan University of Science and Technology, Luoyang, 471023 China; 2 College of Animal Science, Tarim University, Alar, 843300 Xinjiang China; 3 China Agricultural University, Beijing, 100193 China

**Keywords:** *Neospora caninum*, B16F10 melanoma, Th1 immunity, Cell death, Gut microbiota

## Abstract

**Background:**

Melanoma is a malignant tumor with a high mortality rate. Some microorganisms have been shown to activate the immune system and limit cancer progression. The objective of this study is to evaluate the anti-melanoma effect of *Neospora caninum*, a livestock pathogen with no pathogenic activity in humans.

**Methods:**

*Neospora caninum* tachyzoites were inoculated into a C57BL/6 mouse melanoma model by intratumoral and distal subcutaneous injections. Tumor volumes were measured, and cell death areas were visualized by hematoxylin and eosin staining and quantified. Apoptosis in cell cultures and whole tumors was detected by propidium iodide (PI) and TUNEL staining, respectively. Cytokine and tumor-associated factor levels in tumors and spleens were detected by real-time quantitative polymerase chain reaction. Infiltration of macrophages and CD8^+^ T cells in the tumor microenvironment (TME) were detected by immunohistochemistry with anti-CD68 and anti-CD8 antibodies, respectively. Finally, 16S rRNA sequencing of mice cecal contents was performed to evaluate the effect of *N. caninum* on gut microbial diversity.

**Results:**

Intratumoral and distal subcutaneous injections of *N. caninum* resulted in significant inhibition of tumor growth (*P* < 0.001), and more than 50% of tumor cells were dead without signs of apoptosis. *Neospora caninum* treatment significantly increased the mRNA expression levels of IL-12, IFN-γ, IL-2, IL-10, TNF-α, and PD-L1 in the TME, and IL-12 and IFN-γ in the spleen of tumor-bearing mice (*P* < 0.05). An increase in the infiltration of CD8^+^ T cells and macrophages in the TME was observed with these cytokine changes. *Neospora caninum* also restored the abundance of gut microbiota *Lactobacillus*, *Lachnospiraceae*, *Adlercreutzia*, and *Prevotellaceae* associated with tumor growth, but the changes were not significant.

**Conclusion:**

*Neospora caninum* inhibits B16F10 melanoma by activating potent immune responses and directly destroying the cancer cells. The stable, non-toxic, and efficacious properties of *N. caninum* demonstrate the potential for its use as a cancer treatment.

**Graphical Abstract:**

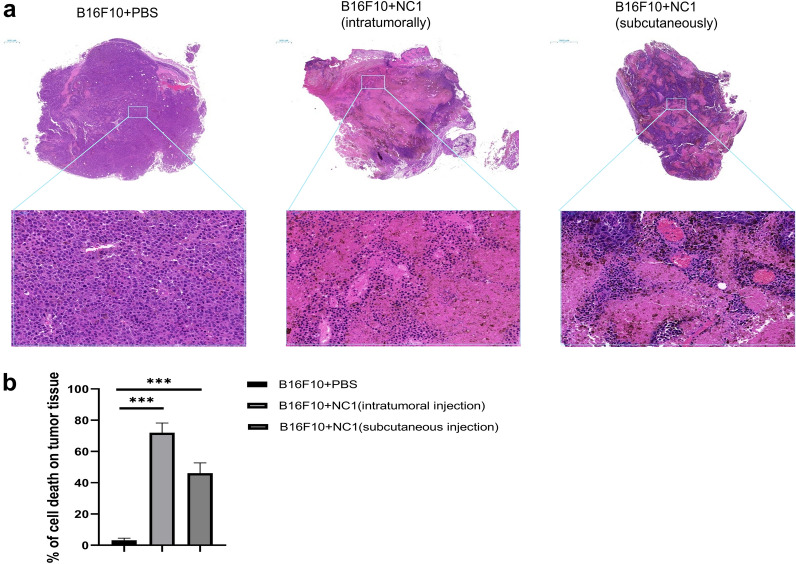

**Supplementary Information:**

The online version contains supplementary material available at 10.1186/s13071-022-05456-8.

## Background

Melanoma is the most aggressive form of skin cancer with a high mortality rate and can give rise to a variety of poorly immunogenic metastases [1]. Immunotherapy plays an increasingly important role in controlling tumorigenesis and progression nowadays. There are now over a dozen immunotherapies approved for cancer treatment, and many more are in clinical trials. These immunotherapies generally fall into several categories: checkpoint inhibitors, lymphocyte-activated cytokines, chimeric antigen receptor (CAR) T cells and other cellular therapies, agonistic antibodies against co-stimulatory receptors, cancer vaccines, oncolytic viruses, and bispecific antibodies [2].

Interestingly, the concept that the immune system can recognize and control tumor growth can be traced back to 1893, when William Coley used live bacteria as an immune stimulant against cancer [3]. While these treatments are generally not acceptable clinically, and relevant studies are rare, the concept of utilizing microorganisms to treat cancer continued to be explored and may hold increasing promise as effective cancer therapies. Morales et al. [4] demonstrated that bacillus Calmette-Guérin (BCG) application could promote cancer regression, and the vaccine was eventually approved as a complementary treatment for bladder cancer. Intralesional injection of *Streptococcus pyogenes* OK-432 (lyophilized culture of human group A *Streptococcus pyogenes*) has been a safe and effective treatment for lymphangiomas in children since 1987 [5], resulting in at least 50% reduction of cyst volume [6]. In addition, some anaerobes and viruses, such as *Clostridium* spp., *Salmonella* spp., *Bifidobacterium* spp., *Listeria* spp., and herpes simplex virus 1 (gene-deficient HSV-1), also have potential to be used as anticancer therapies [7–12].

Another group of microorganisms with potentially interesting antitumor capabilities is parasites, and several studies are beginning to reveal their mechanism of action. Chen et al. [13] found that malaria infections significantly suppressed Lewis’s lung cancer growth via induction of innate and adaptive antitumor responses in mice, suggesting that the malaria parasite may provide a novel strategy or therapeutic vaccine vector for anti-lung cancer immune therapy. Junqueira et al. [14] used a recombinant non-pathogenic clone of *Trypanosoma cruzi* as a vaccine vector to induce strong and long-term T cell-mediated immunity, achieving solid protection against melanoma in mice. Monotherapy with a uracil-deficient strain of *Toxoplasma gondii* could modify the tumor microenvironment (TME) and inhibit tumor growth, including melanoma [15], pancreatic cancer [16], lung cancer [17, 18], and ovarian cancer [19]. Kang et al. [20] found that tumor growth and lung metastasis were significantly reduced in *Trichinella spiralis*-infected mice compared with controls. Thus, these microorganisms may activate the innate immune system to overcome the immunosuppressive TME, resulting in tumor inhibition.

However, microorganisms as immunotherapy have certain disadvantages. They may persist in the body for long periods and become pathogenic over time. Virulence is also an issue; hence, extensive genetic manipulations to reduce virulence are often required to attenuate the microorganisms without compromising their immunostimulatory capacity, allowing them to act as powerful immune adjuvants [7]. Therefore, uncovering new microorganisms that are harmless to humans for therapeutic use is a new and viable endeavor.

The intracellular protozoan parasite *Neospora caninum* is similar to the well-studied apicomplexan parasite *T. gondii* [21], and is a major cause of reproductive failure in dairy cattle worldwide. Although *N. caninum* has been successfully cultured in some human cell lines, and low levels of antibodies against this parasite have been reported in human sera, it has not been demonstrated to cause zoonotic disease [22, 23]. In C57BL/6J mice, *N. caninum* infection was found to induce significant macrophage recruitment to the infection site and increase secretion of interleukin 6 (IL-6), IL-12p40, and interferon gamma (IFN-γ), which was similar to the host immune responses against melanoma [21, 24]. Recently, Lantier et al. [22] demonstrated that intratumoral or distal injection of *N. caninum* tachyzoites strongly inhibited tumor development, and often caused complete eradication, in a murine thymoma model. Therefore, as an antitumor agent, *N. caninum* is safer and more controllable than other Apicomplexa members (*T. gondii* and malaria parasites). As there are no further reports of *N. caninum* against cancer, it is unclear whether *N. caninum* can be used as an adjuvant therapy in melanoma. Therefore, in this study, we evaluated the antitumor activity of *N. caninum* and explored its potential as an immunotherapeutic microorganism in a murine model of B16F10 melanoma, with evaluations of possible mechanisms of action.

## Methods

### Mice

Female C57BL/6 mice (6–7 weeks of age) were purchased from Beijing Vital River Laboratory Animal Technology Co., Ltd. (Beijing, China) and acclimatized for 1 week before use. Animals were housed in suitable rodent facilities with a 12-h photoperiod and provided with continuous standard rodent chow and water. All animal experiments were approved by the Experimental Animal Commission of Henan University of Science and Technology (Permit No. SCXK [JING] 2021–0006). The experimental scheme conformed strictly to the guidelines of the Institutional Animal Care and Use Committee (No. 201) of Henan University of Science and Technology (Luoyang, Henan, China).

### Tumor cell

The B16F10 mouse melanoma cells were purchased from Procell Life Science & Technology (Wuhan, China) and were cultured in RPMI-1640 medium (Servicebio, Wuhan, China) containing 10% fetal bovine serum (FBS; Clark Bio, Shanghai, China) and 100 U/ml of penicillin/streptomycin (Severn Biotech, Beijing, China) in a humidified incubator at 37 °C with 5% CO_2_.

### *Neospora caninum*

*Neospora caninum* (NC1 strain) tachyzoites were a gift from Prof. Qun Liu of China Agricultural University and were cultured in Vero cells in Dulbecco's modified Eagle medium (DMEM; Servicebio, Wuhan, China) containing 10% FBS (Clark Bio, Shanghai, China) in an incubator at 37 °C with 5% CO_2_, as described previously [25]. Parasites were purified by centrifugation at 750 g for 10 min and washed twice in phosphate-buffered saline (PBS, pH = 7.2–7.5) before use.

### Animal experiments and sample collection

**Fig. 1:**
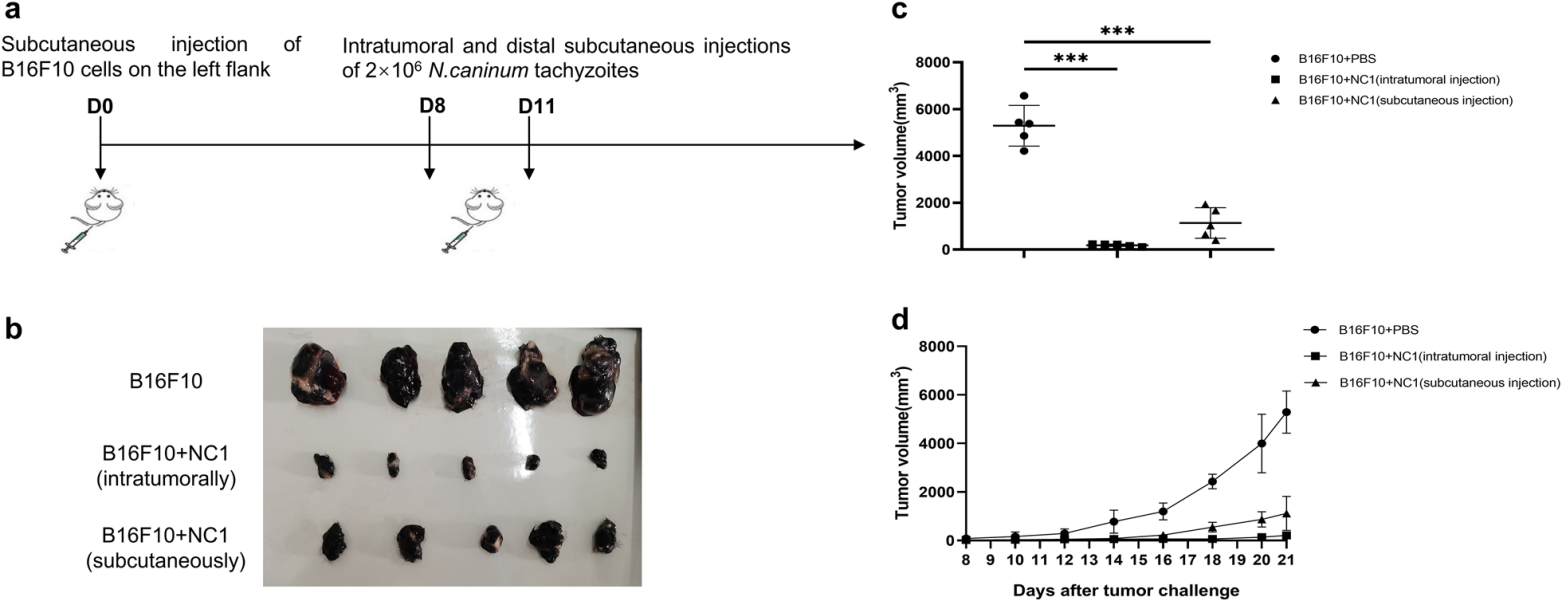
Live *N. caninum* tachyzoites inhibit established B16F10 melanoma in mice. **a** Mice experiment schedule: B16F10 cells (2 × 10^5^) were inoculated subcutaneously on the left flank of C57BL/6 mice, and intratumoral or distal subcutaneous injections of *N. caninum* were given when tumor diameter reached 3–5 mm, at a dose of 2 × 10^6^ tachyzoites per mouse. **b** The macroscopic size of tumors in each group of mice on day 21 (*n* = 5). **c** Quantification of the dissected tumor volume on day 21 for each treatment group (mean ± SD; *n* = 5). **d** Comparison of tumor volume in tumor-bearing mice (*n* = 5) treated with PBS or *N. caninum* tachyzoites. Data were analyzed by the two-tailed Student *t*-test (**c**). *P < 0.05, **P < 0.01, ***P < 0.001. Abbreviations: B16F10 + PBS, tumor control group; intratumorally, intratumoral injection; subcutaneously, distal subcutaneous injection; subcutaneous injection, distal subcutaneous injection

Mice were randomly divided into four groups, except the mice in the blank control group, and all mice were injected subcutaneously with 5 × 10^5^ B16F10 cells suspended in PBS. Tumor sizes were measured every 2 days. When tumor size reached 3–5 mm in diameter, tumor-bearing mice were injected intratumorally (intratumoral *N. caninum* injection group) or subcutaneously in the contralateral flank (distal subcutaneous *N. caninum* injection group) with 2 × 10^6^ *N. caninum* tachyzoites suspended in PBS or PBS (tumor control group), respectively, as outlined in Fig. 1a.

Mice were monitored daily, and the survival end point was determined by either spontaneous death or the presence of moribund signs. Mice were euthanized (carbon dioxide asphyxiation) on day 21 of the tumor challenge, tumor tissues were harvested, and the volume was calculated using the formula *V* = (*ab*^*2*^)/2, where *a* is the long axis and *b* is the short axis of the tumor. Tumor tissues were divided into three parts for histology, immune gene analysis, and *N. caninum* detection by Nc5 PCR [26]. The primers for Nc5 were as follows: F: 5′-CTCGCAGTCAACCTACGTCTTCT-3′; R: 5′-CCCAGTGCGTCCAATCCTGTAAC-3′. Concurrently, the spleen was collected for immune gene analysis and *N. caninum* detection. Other organs including the heart, liver, lung, and brain were used for *N. caninum* detection only. In addition, the cecal contents in each group were collected for gut microbiota analysis.

### Histological analysis

Samples were fixed with 4% paraformaldehyde, paraffin-embedded, and sectioned into 3 μm sections. For overall pathological evaluation, samples were stained with hematoxylin and eosin (H&E) and assessed with light microscopy. Tumor-infiltrating CD8^+^ T cells were detected with anti-CD8 antibody (Servicebio, Wuhan, China), and macrophages with anti-CD68 (Servicebio, Wuhan, China), according to the manufacturer's instructions. Stained tumor sections were imaged with a digital scanner, and the tumor area, number of positive cells, and total cells were quantified by the Servicebio image analysis system.

### Real-time quantitative polymerase chain reaction

Total RNA was isolated from homogenized tissue with the FastPure Cell/Tissue Total RNA Isolation Kit V2 (Vazyme, Nanjing, China) and reverse-transcribed with the HiScript III RT SuperMix for qPCR (Vazyme, Nanjing, China) according to the protocol provided by the manufacturer. Real-time quantitative PCR (RT-PCR) for IL-2, IL-10, IL-12, IL-15, tumor necrosis factor alpha (TNF-α), IFN-γ, programmed death-ligand 1 (PD-L1), VEGF-A, and hypoxia-inducible factor 1-alpha (HIF-1α) was performed using the PerfectStart™ Green qPCR SuperMix (Transgen, Beijing, China) in a 7500 Fast Real-Time qPCR System (Bio-Rad, Hercules, CA, USA) with three replicates. Primer sequences are shown in Additional file 1: Table S1. Relative gene expression was quantified with the 2^–△△Ct^ method. Data from triplicate experiments are presented as the fold difference in gene expression normalized to β-actin.

### Detection of tumor cell apoptosis in vivo and in vitro

Tumor cell death in vivo was detected via terminal deoxynucleotidyl transferase dUTP nick end labeling (TUNEL; Servicebio, Wuhan, China) staining of tumor tissue sections. In vitro, cell death was evaluated by infecting B16F10 cells with a 1:2 multiplicity of infection (MOI) of *N. caninum* tachyzoites, followed by staining with the YO-PRO-1/PI dual staining Apoptosis and Necrosis Detection Kit (Beyotime, Shanghai, China) at 2, 6, 18, 30, and 42 h post-infection, according to the manufacturer’s instructions. Images were captured using a fluorescence microscope (Zeiss Axio Observer A1, Oberkochen, Germany).

### Gut microbiota analysis

Contents of the cecum were collected, and total DNA (~ 100 mg) was extracted for 16 s rRNA sequencing. Paired-end (200–300 bp) sequencing was performed on the Illumina MiSeq system (Illumina, San Diego, CA, USA) with barcoded purified amplicons normalized in equimolar amounts, according to standard protocols by Majorbio Bio-Pharm Technology (Shanghai, China).

### Statistical analysis

Statistical analysis was performed using the GraphPad Prism 8.0 software. The two-tailed Student *t*-test and the Mann–Whitney test were used to analyze parametric and non-parametric data, respectively. Differences of *P* < 0.05 were considered to be statistically significant.

## Results

### *Neospora caninum* live tachyzoites inhibit and regress established B16F10 melanoma in mice

To determine whether *N. caninum* can suppress melanoma development, we treated mice with established B16F10 tumors (3–5 mm) with live *N. caninum* tachyzoites either intratumorally or distal subcutaneously on the contralateral flank (Fig. 1a). On day 21, one mouse from the tumor control group died, and five mice from each group were randomly selected for euthanasia. After two doses of tachyzoites, given 3 days apart, we observed a significant reduction in tumor volume (*t*-test, *t*_(8)_ = 13.11, *P* < 0.001 for intratumoral injection; *t*_(8)_ = 8.55, *P* < 0.001 for subcutaneous injection), regardless of whether the injection was made intratumorally or at a distant site (Fig. 1b, c), and greatly retarded tumor growth (Fig. 1d), without any effect on the body weight of mice (Additional file 2: Fig. S1).

Tumor-bearing mice treated with *N. caninum* did not develop any adverse events, even though *N. caninum* DNA could be detected only in the lungs, brain, and tumor 13 days post-treatment. In vitro, *N. caninum* could infect and replicate within B16F10 cells, demonstrating the replication ability of *N. caninum* in host cells (Additional file 3: Fig. S2).

### Cell death in B16F10 melanoma tissue after treatment with *N. caninum*

**Fig. 2:**
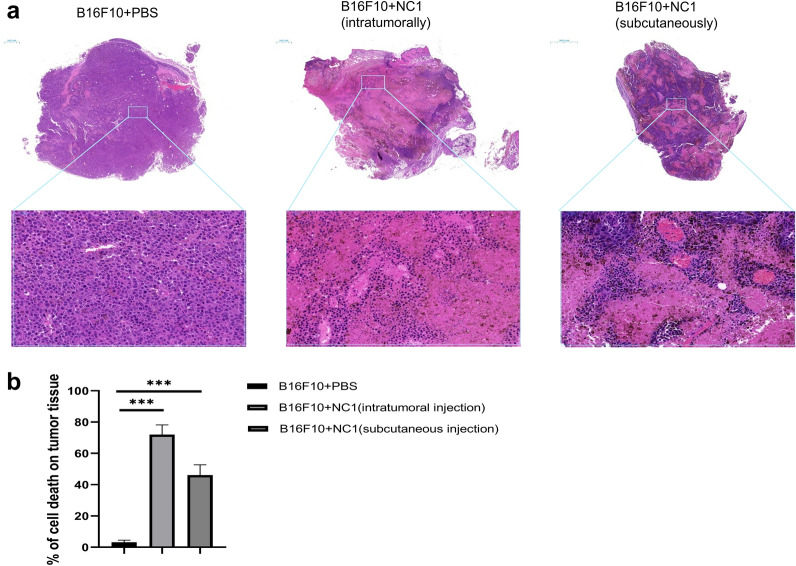
*Neospora caninum* treatment induces tumor cell death. **a** Tumors harvested on day 21 after tumor inoculation from mice treated with PBS or *N. caninum* tachyzoites were stained with H&E. The top row shows the entire tumor area; the bottom row shows enlargement of a section of the tumor bed; bars = 500 μm and 50 μm. **b** Quantification of the percentage of cell death areas for each treatment group (mean ± SD; *n* = 5). Data were analyzed by the two-tailed Student *t*-test (**b**). *P < 0.05, **P < 0.01, ***P < 0.001

H&E staining of tumor sections revealed almost no tumor cell death in untreated tumors (cell death area = 3.2% of the tumor area), while large areas of dead cells were observed in mice treated with *N. caninum*. The cell death area was 72.1% for the intratumorally injected group and 46.2% for the distal subcutaneously injected group (Fig. 2a, b).

### *Neospora caninum* induces tumor cell death but not via apoptosis

**Fig. 3:**
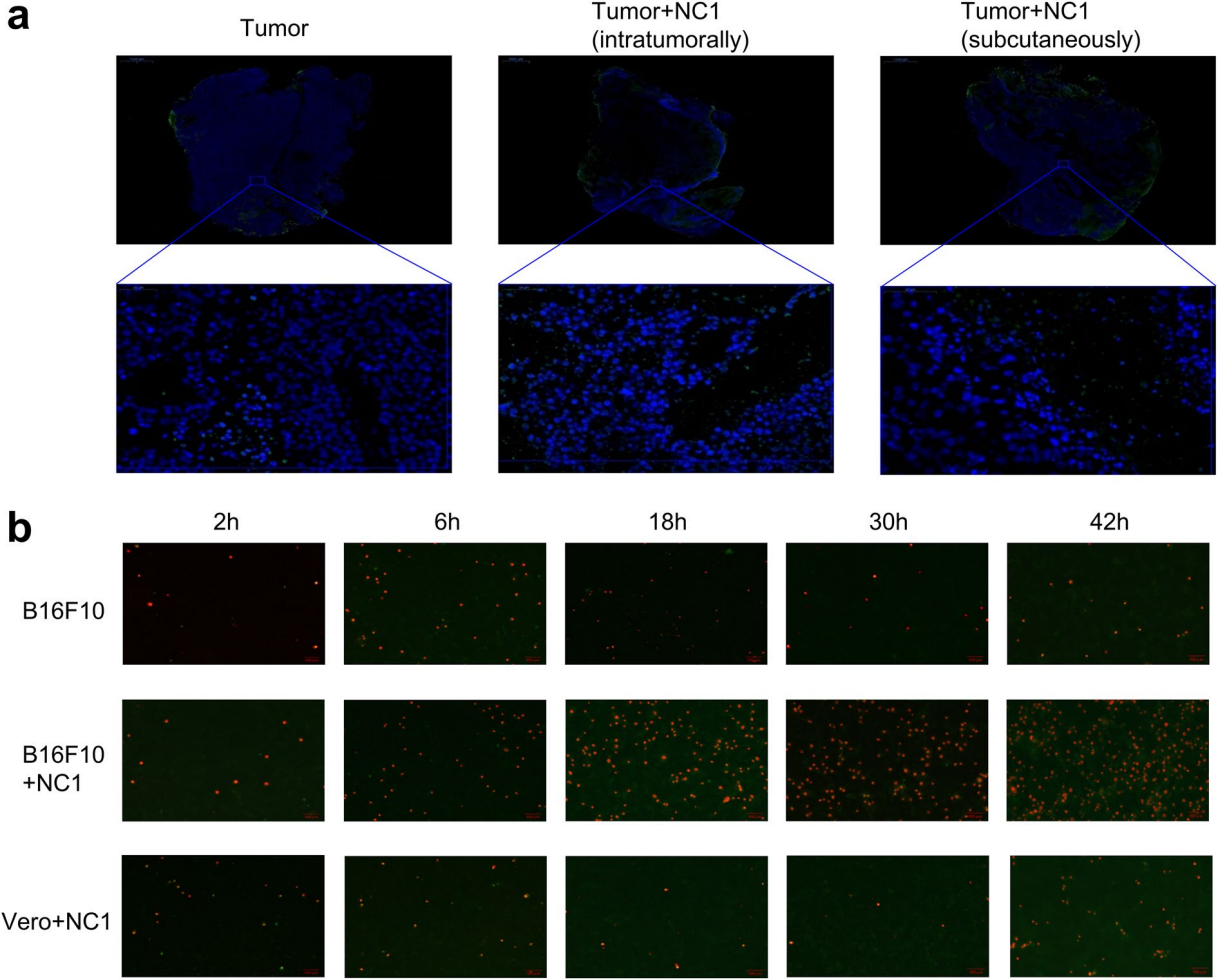
*Neospora caninum* induces tumor cell death but not via apoptosis. **a** Apoptotic cells (green) were detected by TUNEL assay in tumor tissue of mice treated with or without *N. caninum*; nuclei (blue) were stained with DAPI. The top row shows the entire tumor area (merge); the bottom row shows enlargement of a section of the tumor bed; bars = 1000 μm and 50 μm, respectively. **b** Detection of apoptosis (YO-PRO-1; bright green) and necrosis (double stained with PI, bright red; YO-PRO-1, weak green) in B16F10 cells and Vero cells at 2, 6, 18, 30, and 42 h after infection with or without *N. caninum*. Magnification ×100. Bar = 100 μm

To determine whether the observed in vivo cell death involves apoptotic pathways, we first stained the tumor sections with the TUNEL assay. Fluorescence TUNEL signals were very low or undetectable in all tumor samples, and no significant difference could be observed between treatment groups, suggesting that *N. caninum* did not cause apoptosis in B16F10 melanoma (Fig. 3a). To confirm that apoptosis is indeed not involved, we treated B16F10 cells with *N. caninum* in vitro and harvested cells at different time points for apoptosis and necrosis detection. Double staining for apoptosis (YO-PRO-1; green) and necrosis markers (PI; red) showed almost no green fluorescence at all time points post-infection, and substantial increase in red fluorescence after 6 h, indicating that *N. caninum* induces tumor cell death, but not via apoptosis (Fig. 3b).

### *Neospora caninum* promotes production of Th1 cytokines

**Fig. 4:**
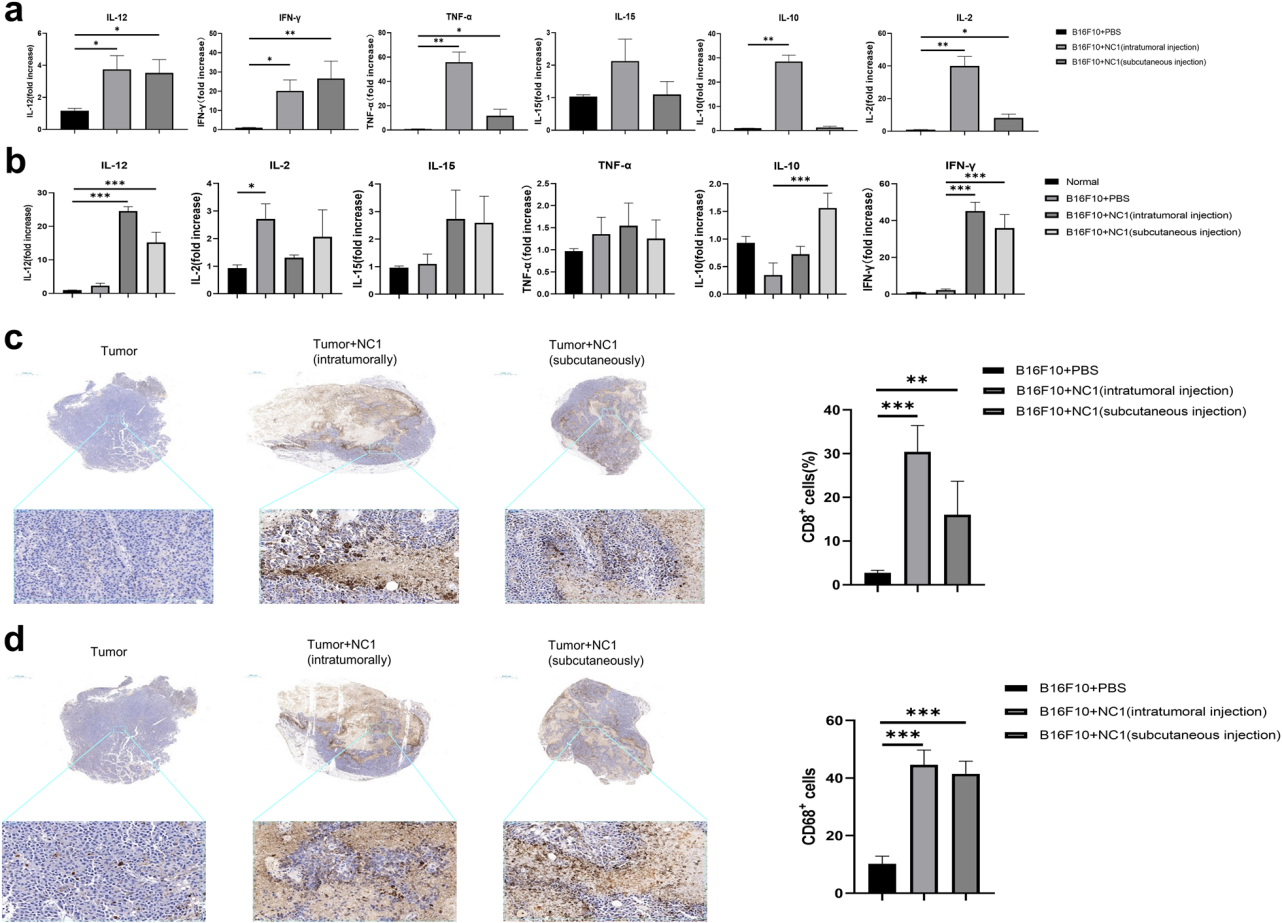
*Neospora caninum* infection induces Th1 cytokine production and increases infiltration of CD8^+^ T cells and macrophages into the tumor microenvironment. Twenty-one days after B16F10 cells inoculation, cytokine expression in tumors (**a**) and spleens (**b**) were detected by RT-PCR analysis. Graphs show fold change relative to control (mean ± SD; *n* = 5). CD8^+^ T cells (**c**) and macrophages (**d**) in the tumor microenvironment were stained with anti-CD8 and anti-CD68 (brownish yellow), respectively; the nuclei (blue) were stained with hematoxylin. The top row shows the entire tumor area; the bottom row shows enlargement of a section of the tumor bed; bars = 1000 μm and 50 μm, respectively. The graph shows the quantitative analysis of CD8^+^ and CD68^+^ cells (mean ± SD; n = 5). Data were analyzed by the two-tailed Student *t*-test (**a–d**). *P < 0.05, **P < 0.01, ***P < 0.001. Abbreviations: normal, blank control group

We evaluated the tumor and spleen by RT-PCR to determine the cytokine changes in mice. Compared with mice in the tumor control group, intratumoral *N. caninum* significantly increased the levels of IL-12 (*t*-test, *t*_(8)_ = 3.11, *P* = 0.014), IFN-γ (*t*-test, *t*_(8)_ = 3.28, *P* = 0.011), IL-2 (*t*-test, *t*_(8)_ = 4.62, *P* = 0.002), IL-10 (*t*-test, *t*_(8)_ = 4.29, *P* = 0.003), and tumor necrosis factor-α (TNF-α) (*t*-test, *t*_(8)_ = 4.98, *P* = 0.001) in the tumor; IL-15 was increased but not statistically significant (*t*-test, *t*_(8)_ = 2.25, *P* = 0.054) (Fig. 4a). Interestingly, distal injection of *N. caninum* also had similar effects to intratumoral injection, particularly for IL-12 (*t*-test, *t*_(8)_ = 2.69, *P* = 0.002) and IFN-γ (*t*-test, *t*_(8)_ = 4.62, *P* = 0.002) where similar levels were induced. The levels of IL-2 (*t*-test, *t*_(8)_ = 3.23, *P* = 0.012) and TNF-α (*t*-test, *t*_(8)_ = 3.23, *P* = 0.012) were much lower, but still significantly above control, while IL-10 (*t*-test, *t*_(8)_ = 1.72, *P* = 0.137) and IL-15 (*t*-test, *t*_(8)_ = 0.58, *P* = 0.577) levels were not significantly affected (Fig. 4a). In the spleen, IL-12 (*t*-test, *t*_(8)_ = 24.08, *P* < 0.0001 for intratumoral injection; *t*_(8)_ = 12.50, *P* < 0.0001 for subcutaneous injection) and IFN-γ (*t*-test, *t*_(8)_ = 20.08, *P* < 0.0001 for intratumoral injection; *t*_(8)_ = 11.05, *P* < 0.0001 for subcutaneous injection) were significantly elevated in both intratumoral and distal injection groups; IL-10 was significantly elevated in the distal subcutaneous injection group (*t*-test, *t*_(8)_ = 7.04, *P* = 0.0001); IL-15 (*t*-test, *t*_(8)_ = 1.57, *P* = 0.156 for intratumoral injection; *t*_(8)_ = 1.52, *P* = 0.168 for subcutaneous injection) and TNF-α (*t*-test, *t*_(8)_ = 0.43, *P* = 0.681 for intratumoral injection; *t*_(8)_ = 0.28, *P* = 0.787 for subcutaneous injection) were increased but the difference was not statistically significant. The levels of IL-2 tended to decrease, but again not statistically significant (*t*-test, *t*_(8)_ = 2.11, *P* = 0.068 for intratumoral injection; *t*_(8)_ = 1.78, *P* = 0.113 for subcutaneous injection) (Fig. 4b). In line with the increase in Th1 cytokines, we observed significant increase in CD8^+^ T cells (*t*-test, *t*_(8)_ = 10.30, *P* < 0.0001 for intratumoral injection; *t*_(8)_ = 3.84, *P* = 0.005 for subcutaneous injection) and CD68^+^ macrophages (*t*-test, *t*_(8)_ = 13.41, *P* < 0.0001 for intratumoral injection; *t*_(8)_ = 13.69, *P* < 0.0001 for subcutaneous injection) in the tumors (Fig. 4c, d).

**Fig. 5:**
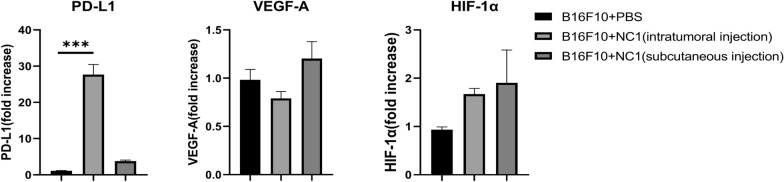
Analysis of immunosuppressive factors in tumor tissues. Twenty-one days after inoculation with B16F10 cells, immunosuppressive factors in tumors were detected by RT-PCR. Graphs show fold change relative to control (mean ± SD; *n* = 5). Data were analyzed by the two-tailed Student *t*-test. *P < 0.05, **P < 0.01, ***P < 0.001

Detection of common immunosuppressive factors showed that PD-L1 levels were significantly higher after intratumoral injection of *N. caninum* compared with the tumor control group (*t*-test, *t*_(8)_ = 24.17, *P* < 0.0001); VEGF-A (*t*-test, *t*_(8)_ = 1.91, *P* = 0.083) and HIF-1α (*t*-test, *t*_(8)_ = 2.22, *P* = 0.057) were elevated but not statistically significant. Distal injection of *N. caninum* also did not affect these proteins significantly (*t*-test, *t*_(8)_ = 2.25, *P* = 0.065, *t*_(8)_ = 0.87, *P* = 0.409, and *t*_(8)_ = 2.08, *P* = 0.071 for PD-L1, VEGF-A, and HIF-1α, respectively) (Fig. 5).

### Effect of *N. caninum* on the gut microbiota of tumor-bearing mice

**Fig. 6:**
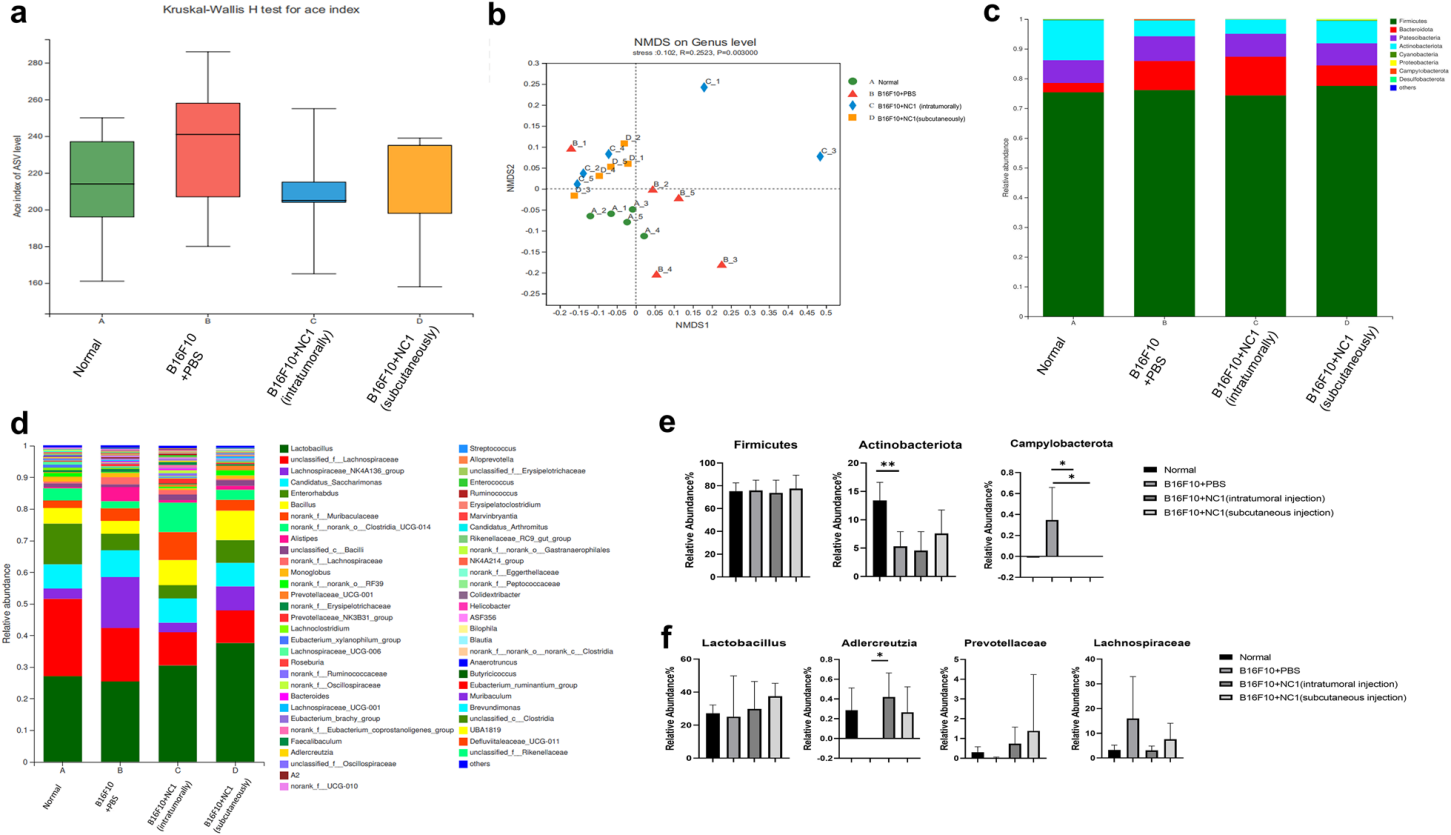
*Neospora caninum* treatment alters gut microbiota composition. **a** A Kruskal–Wallis *H*-test was performed to compare alpha microbial diversity indices and community richness among the four groups. NMDS analyses (**b**) reveal significant differences in the gut microbiota of mice with and without *N. caninum* treatment based on the weighted UniFrac distances at the genus level. Relative abundance of gut microbiota at the phylum (**c**) and genus (**d**) level. Selected direct comparison at the phylum level for Firmicutes, Actinobacteria, and Campylobacterota (**e**), and at the genus level for *Lactobacillus*, *Lachnospiraceae*, *Adlercreutzia*, and *Prevotellaceae* (**f**); graphs show relative abundance (mean ± SD; *n* = 5). Data were analyzed by the two-tailed Student *t*-test (**e, f**), *P < 0.05, **P < 0.01, ***P < 0.001

The gut microbiome is integral to health and disease. *Neospora caninum* may interact with the gut microbiota to affect host immune response against cancer. Hence, we performed 16S rRNA sequencing on mouse cecum to determine the effect of *N. caninum* on microbial diversity. The amplicon sequence variant (ASV) α-diversity index was found to be higher in tumor-bearing mice than in the PBS group. *Neospora caninum* treatment restored the ASV α-diversity index, although the differences among groups were not significant (*P* = 0.607) (Fig. 6a). In addition, beta diversity was used to determine differences in the overall composition of the microbial community of the groups. Non-metric multidimensional scaling (NMDS) analysis at the genus level showed significant changes in the diversity of the gut microbiota in the tumor group versus the PBS group (*R* = 0.252, *P* = 0.003). The two *N. caninum*-treated groups showed significant divergency from the tumor-only group, but did not coincide with the PBS group, and no significant difference was observed between injection sites (Fig. 6b), suggesting that *N. caninum* treatment induces unique changes to the gut microbiota. A closer look revealed the predominant bacteria at the phylum level were Firmicutes and Bacteroidetes followed by Patescibacteria and Actinobacteria, with tumor induction resulting in increased Bacteroidetes and decreased Actinobacteria populations. Interestingly, intratumoral and distal injections of *N. caninum* did not alter the gut microbiota in the same way: the intratumoral group showed a further increase in Bacteroidetes and no change to Actinobacteria compared with tumor-only mice; the distally injected mice showed reduced Bacteroidetes and increased Actinobacteria (Fig. 6c). Tumor development significantly increased Campylobacterota abundance compared with the PBS group, and the normal level was restored by *N. caninum* treatment (Fig. 6e). At the genus levels, *Lactobacillus*, *Lachnospiraceae,* and *Candidatus Saccharimonas* were the three major genera in all mice (Fig. 6d). Compared with the B16F10 group, treatment with *N. caninum* increased the relative abundance of *Lactobacillus* (*t*-test, *t*_(8)_ = 0.36, *P* = 0.729 for intratumoral injection; *t*_(8)_ = 1.07, *P* = 0.316 for subcutaneous injection)*, Adlercreutzia* (*t*-test, *t*_(8)_ = 2.79, *P* = 0.024 for intratumoral injection; *t*_(8)_ = 2.28, *P* = 0.052 for subcutaneous injection)*,* and *Prevotellaceae* (*t*-test, *t*_(8)_ = 1.86, *P* = 0.101 for intratumoral injection; *t*_(8)_ = 1.06, *P* = 0.320 for subcutaneous injection) and decreased the relative abundance of *Lachnospiraceae* (*t*-test, *t*_(8)_ = 1.69, *P* = 0.130 for intratumoral injection; *t*_(8)_ = 1.03, *P* = 0.333 for subcutaneous injection)*,* but the changes were not significant (Fig. 6f).

## Discussion

The use of microorganisms as immune stimulants appears to be one of the most original strategies in known and practiced anticancer therapies [27]. Patients who have failed conventional therapies have a greater likelihood of recovery with microbial treatment; these approaches are more selective and induce less adverse effects in patients as a whole [27, 28]. However, the use of microorganisms in cancer therapy is often overlooked. Currently, there are few reported studies of utilizing parasites for cancer treatment [13–15, 20, 22, 29]. This study provides the first example of effective immunotherapy using live *N. caninum* in a mouse model of B16F10 melanoma, which promoted the production of Th1 cytokines in the tumor and spleen, induced extensive tumor cells death, inhibited the growth of B16F10 melanoma, and prolonged the survival of tumor-bearing mice.

It has been documented that promoting tumor apoptosis is the main reason of some pathogens or drugs inhibiting tumor growth [13, 30]. However, in this study, the results indicated that *N. caninum* induces tumor cell death, but not via apoptosis. The death pathway of tumor cells will be investigated in future experiments.

We observed that both distal and local intratumoral injection of *N. caninum* were able to induce tumor death with varying degrees of success, suggesting at least two possible antitumor mechanisms: first, the distal subcutaneous injection may activate a systemic antitumor immune response, and second, intratumoral injection induced significantly more cell death (Fig. 2), suggesting a direct tumorolytic effect for *N. caninum.*

Tumors are generally non-immunogenic; transforming a non-inflammatory TME (cold tumors) to an inflammatory TME (hot tumors) is one of the key strategies in current anticancer immunotherapy [31–34]. Oncolytic virotherapy is promising in this regard with its ability to trigger potent antitumor immune responses [35–37]. CD8^+^ T cells are considered to be the main driver of antitumor immunity [38], but intra-tumor CD8^+^ T cells are rare and often exhausted due to long-term suppression by the tumor [39]. In this study, we showed that *N. caninum* injection increased infiltration of CD8^+^ T cells and macrophages into the tumor, along with increased expression of IL-12, IFN-γ, IL-10, TNF-α, and IL-2 mRNA, which can also be induced by *N. caninum* in a mouse model, as reported in other studies [21, 24]. This strong induction of the adaptive immune response may resist the immunosuppressive TME, suggesting that *N. caninum* can also convert ''cold tumors'' to ''hot tumors.''

Both treatment regimens in this study showed significant increase in IL-12 and IFN-γ levels in the tumor and spleen. Interleukin-12 has multiple immune effects and is a representative cytokine that triggers antitumor activity [40], stimulating T, natural killer (NK), and natural killer T (NKT) cells to secrete various cytokines, especially IFN-γ, and promote Th1 responses [41–43]. In addition, IL-12 can activate mechanisms that inhibit angiogenesis, thus inhibiting tumor development [44, 45]. Interleukin-10 on the other hand, was previously thought to be largely immunosuppressive and supportive of tumor growth [46, 47]. However, increasing in vivo evidence points to an immunostimulatory role for IL-10 in mediating the antitumor activity of CD8^+^ T cells [39, 48, 49], including enhancing proliferation of IL-2- and IL-4-activated CD8^+^ T cells and rescuing T cells from apoptosis [50, 51]. Mumm et al. [39] further demonstrated that IL-10 induces IFN-γ and granzyme production in CD8^+^ T cells, resulting in increased intra-tumor antigen presentation. Our results also support an antitumor role for IL-10 (Fig. 4b).

We analyzed several common immunosuppressive factors and found significant upregulation of PD-L1 after *N. caninum* treatment. This may be caused by elevated levels of IFN-γ within the tumor. It has been shown that subcutaneous injection of IFN-γ could induce PD-L1 expression and promote tumor growth; the effect was abrogated in PD-L1-depleted mice [52]. Our results contrast the report by Lantier et al. [22], where *N. caninum* treatment reduced PD-L1 levels in EG-7 tumors. However, Zhu et al. [53] found that a non-toxic ΔGRA17 mutant *Toxoplasma gondii* strain could induce B16F10 melanoma regression, with significant elevation of PD-L1 in the TME. Combination therapy with the mutant *T. gondii* strain and anti-PD-L1 further arrested melanoma growth and significantly improved survival. Thus, different tumor types and parasites may give rise to different observations.

We showed that *N. caninum* induced the death of B16F10 cells in vitro, but not via apoptosis (Fig. 3b). H&E staining of sections from the intratumoral injection group showed an increased number of tumor cell death caused by *N. caninum*. Therefore, we hypothesize that *N. caninum* may directly lyse tumor cells. This is consistent with a previous report by Lantier et al. [22] showing decreased tumor volume in an *N. caninum*-treated non-obese diabetic/severe combined immunodeficiency (NOD/SCID) model of human Merkel cell carcinoma (MCC). In addition, we speculate that the role of *N. caninum* in tumor cells may be similar to that of oncolytic virus, increasing inflammatory cytokines and immune cell infiltration in the TME.

In mice treated with distal subcutaneous injection of *N. caninum*, despite the absence of *N. caninum* in the tumor, a strong Th1 immune response was observed in the TME. Moreover, *N. caninum* did not express any tumor antigens, but inhibited tumor growth. We hypothesize that distal subcutaneous injection of *N. caninum* can disrupt immunosuppression and activates innate immune function, thereby supporting an existing but suppressed adaptive immune response. The tumor growth curves showed that tumor growth slowed and tended to shrink after two distal subcutaneous injections, but the tumors tended to regrow after cessation of *N. caninum* injection, indicating that the tumor may again overcome the immune response.

In clinical treatment of tumors, it is often difficult to treat with intratumoral injections, while distal subcutaneous injections are a more convenient and faster treatment method. These results demonstrate the potential of distal subcutaneous injection of *N. caninum* for treatment and deserve further study.

The toxicity profile of potential therapeutic microorganisms is critical to ensure patient safety; efficacy is achieved by a balance between virulence of the microorganism and the strength of the immune response it activates [27]. There is no direct evidence that neosporosis caused by *N. caninum* is a zoonotic disease [54]. In this study, *N. caninum* DNA could only be detected in the lungs and brain of tumor-bearing mice at day 13 post-treatment, without any adverse events. Lantier et al. [22] also showed that *N. caninum* was undetectable in tumors or peripheral organs such as the spleen, brain, and liver 18 days post-treatment. *Neospora caninum* is safe for humans, and may cause neosporosis in some immunocompromised animals when used as an anticancer treatment. However, *N. caninum* can be killed with some drugs (sulfonamides for example), so it is controllable during oncology treatment [22, 55]. Thus, *N. caninum* may be a safe microbial anticancer therapy. In addition, *N. caninum* tachyzoites are very stable and easy to culture in mammalian cells for multiple injections. The risk of mutagenesis and genome integration is also low [22], which is an additional favorable feature.

An increasing number of studies have shown that gut microbes can modulate the host immune system and thus play a role in cancer immunotherapy [56–58]. Our results suggest that *N. caninum* could enhance host systemic and antitumor immunity, but it is unclear whether *N. caninum* achieved immune enhancement indirectly by affecting host gut microbes. In this study, we showed that *N. caninum* treatment increased the abundance of the probiotic *Lactobacillus* (Fig. 5f). Recent studies have shown that *Lactobacillus* can enhance antitumor immunity by promoting Th1 response, CD8^+^ T cell activity, and NK cell infiltration in a colon cancer model [59]. Probiotics can also enhance host immunity and inhibit tumor growth through tumor immunomodulation [60]. In addition, *N. caninum* treatment seemingly restored the abundance of *Lachnospiraceae*, *Adlercreutzia*, and *Prevotellaceae* to normal levels, although the changes were not statistically significant. The possible role of these microorganisms in melanoma development and eradication warrants further study.

## Conclusions

In summary, results in this study revealed that the protozoan *N. caninum* showed strong efficacy against mouse B16F10 melanoma by activating potent immune responses and inducing tumor cell death. Additionally, the stability, safety, and facile culture characteristics of *N. caninum* suggest that it can be developed into a valuable tool for clinical application against cancer.

## Supplementary Information

**Additional file 1: Table S1. **The primers used for RT-PCR.

**Additional file 2: Figure S1. **Evolution of body weight in tumor-bearing mice (*n* = 5) treated with PBS or *N. caninum* tachyzoites.

**Additional file 3: Figure S2. **Visualization of *N. caninum*-GFP tachyzoites in B16F10 cells.

## Data Availability

The data sets supporting the findings of this article are included within the article.

## Abbreviations

TME: Tumor microenvironment
BCG: Bacillus Calmette-Guérin
IFN-γ: Interferon gamma
H&E staining: Hematoxylin and eosin staining
TNF-α: Tumor necrosis factor alpha
NMDS: Non-metric multidimensional scaling
MCC: Merkel cell carcinoma
RT-PCR: Real-time quantitative polymerase chain reaction
