# Supplementary material for: Neospora caninum inhibits tumor development by activating the immune response and destroying tumor cells in a B16F10 melanoma model
Source: Parasit Vectors. 2022 Sep 23;15:332. doi: 10.1186/s13071-022-05456-8 (PMC9503190; doi:10.1186/s13071-022-05456-8)
Supplement: Supplementary file 1 — Additional file 1: Table S1. The primers used for RT-PCR. [file 13071_2022_5456_MOESM1_ESM.docx]

| Gene | Primers | Sequences（5′→3′） |
| --- | --- | --- |
| β-actin | β-actin-F | CCACGAAACTACCTTCAACTCC |
|  | β-actin-R | GTGATCTCCTTCTGCATCCTGT |
| PD-L1 | PD-L1-F | ACTTGCTACGGGCGTTTACT |
|  | PD-L1-R | CTCTCCCCCTGAAGTTGCTG |
| VEGF-A | VEGF-A-F | TTCGTCCAACTTCTGGGCTC |
|  | VEGF-A-R | ACAGCAGTAAAGCCAGGGTC |
| FOXP3 | FOXP3-F | CACCTATGCCACCCTTA CCG |
|  | FOXP3-R | CATGCGAGTAAACCAATGGTAGA |
| HIF-1a | HIF-1a-F | TTGACAAGCTAGCCGGAGGA |
|  | HIF-1a-R | GGGGAAGTGGCAACTGATGA |
| IFN-γ | IFN-γ-F | GCTTTGCAGCTCTTCCTCATG |
|  | IFN-γ-R | TCTTCCACATCTATGCCACTTGA |
| IL-12P40 | IL-12-F | TGGTTTGCCATCGTTTTGCTG |
|  | IL-12-R | ACAGGTGAGGTTCACTGTTTCT |
| IL-2 | IL-2-F | CGGCATGTTCTGGATTTGACT |
|  | IL-2-R | TCATCATCGAATTGGCACTCA |
| IL-4 | IL-4-F | TTGAACGAGGTCACAGGAGAAG |
|  | IL-4-R | CCTTGGAAGCCCTACAGACG |
| IL-10 | IL-10-F | GCTCTTACTGACTGGCATGAG |
|  | IL-10-R | CGCAGCTCTAGGAGCATGTG |
| TNF-α | TNF-α-F | AAGGGAGAGTGGTCAGGTTGCC |
|  | TNF-α-R | CCTCAGGGAAGAGTCTGGAAAGG |
| IL-15 | IL-15-F | ATGTTCATCAACACGTCCTGACT |
|  | IL-15-R | GCAGCAGGTGGAGGTACCTTAA |

Supplement table 1. Primer sequences for real-time qPCR
