# Supplementary figures and images for: Neospora caninum inhibits tumor development by activating the immune response and destroying tumor cells in a B16F10 melanoma model
Source: Parasit Vectors. 2022 Sep 23;15:332. doi: 10.1186/s13071-022-05456-8 (PMC9503190; doi:10.1186/s13071-022-05456-8)

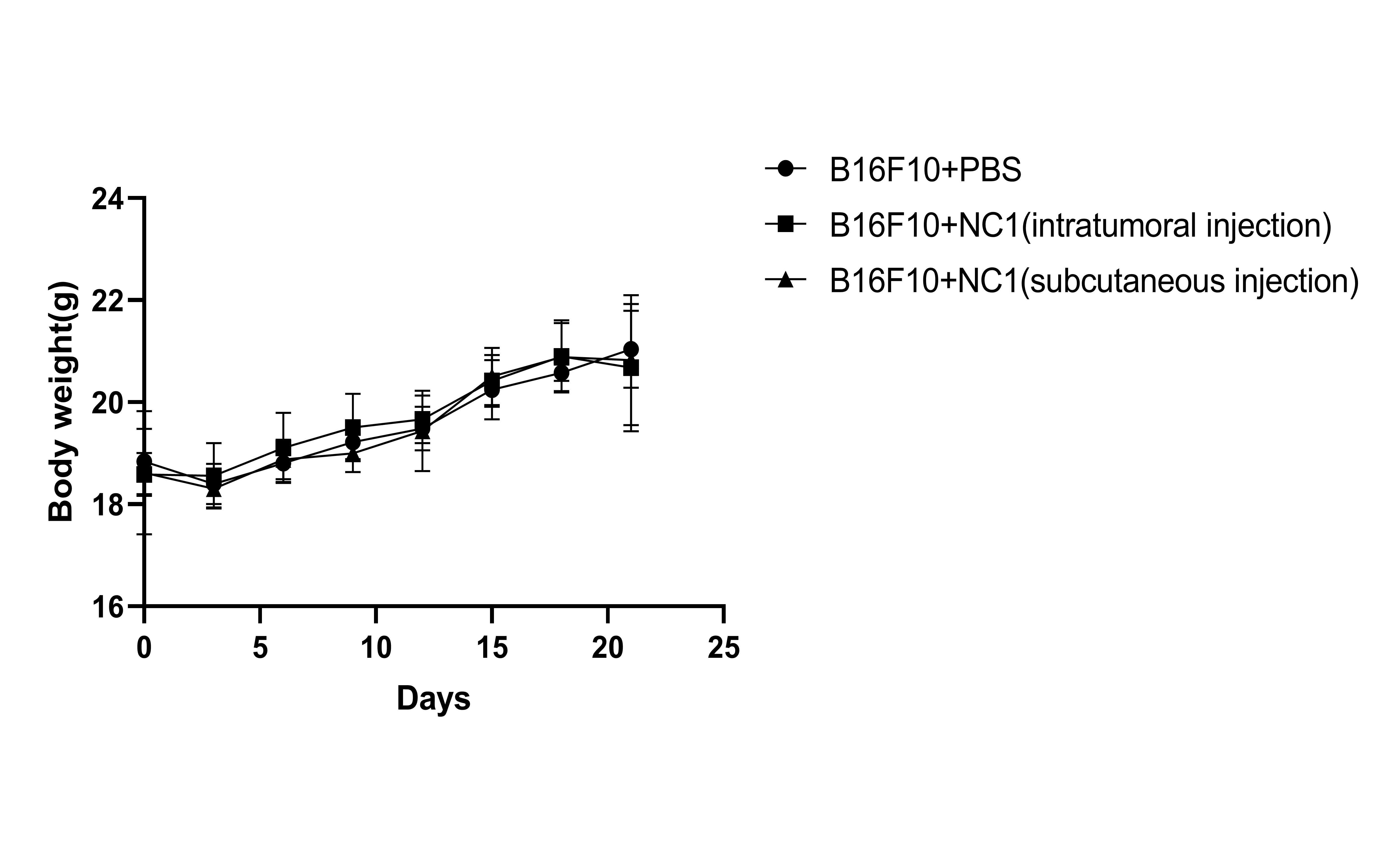

Supplement: Supplementary file 2 — Additional file 2: Figure S1. Evolution of body weight in tumor-bearing mice (n = 5) treated with PBS or N. caninum tachyzoites. [file 13071_2022_5456_MOESM2_ESM.tif]

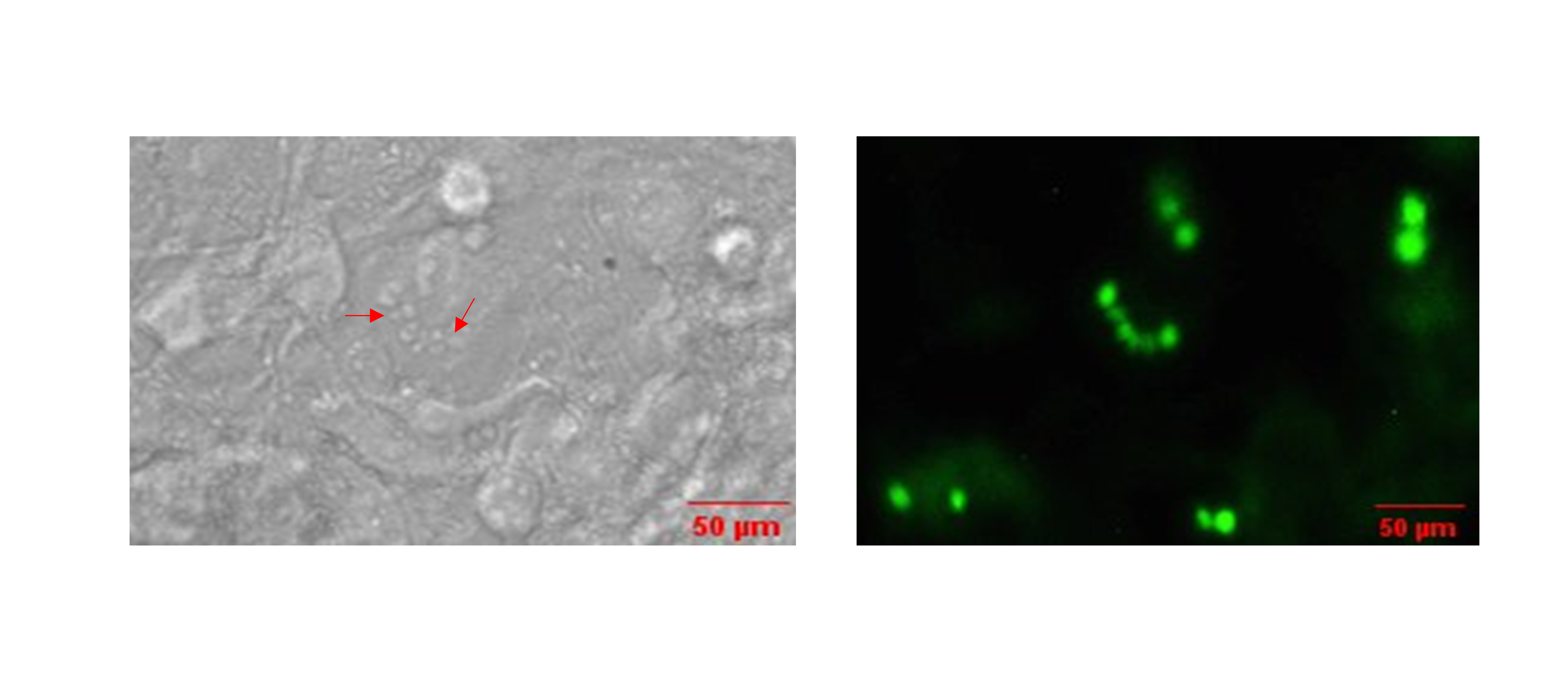

Supplement: Supplementary file 3 — Additional file 3: Figure S2. Visualization of N. caninum-GFP tachyzoites in B16F10 cells. [file 13071_2022_5456_MOESM3_ESM.tif]
